# Transfer Effect of Cognitive Advantages in Visual Working Memory Capacity: Evidence from Elite Football Players

**DOI:** 10.3390/bs13060464

**Published:** 2023-06-02

**Authors:** Xiaomei Wang, Zhigang Liu, Huanyu Zhang, Chaoxin Ji

**Affiliations:** 1 Physical Education Department, Northeastern University, Shenyang 110819, China; 2 Aviation Physical Education Department, Aviation University of Air Force, Changchun 130022, China; 18686515044@163.com (Z.L.); 13944115555@163.com (H.Z.)

**Keywords:** visual working memory capacity, elite football players, novices, cognitive advantages, transfer effect

## Abstract

Background: The research has indicated that elite football players demonstrate cognitive advantages in visual working memory capacity (VWMC); however, it remains unclear whether this effect transfers to other domains cognitive advantages. Object: This study investigated the VWMC differences between elite football players and novices, with a particular focus on cognitive advantages. Methods: Elite football players (specialized in football) and novices were selected to complete the VWMC test task under three different stimulus conditions, then the differences in the VWMCs of elite football players and novices were analyzed. Results: In comparison to novices, elite football players demonstrated cognitive advantages in VWMCs, along with a possible transfer effect. Additionally, the study showed that the reaction times among elite football players and novices differed, with elite players demonstrating shorter reaction times, which is a difference that was amplified as the number of stimuli increased. Conclusion: The VWMCs of elite football players was better than that of novices under professional and meaningless conditions, which indicates that the VWMCs of elite football players has a transfer effect. Through further analysis of the reaction times cognitive advantages, it was found that there are significant differences between elite football players and novices when responding to the stimuli in both professional and meaningless conditions.

## 1. Introduction

Working memory is a limited capacity storage system for short-term storage and processing of information [1]. Baddeley’s core model suggests that working memory is composed of a visual–spatial sketch pad, central executive, and phonological loop [2]. In recent decades, working memory has been the focus of researchers’ attention. Many researchers have investigated the measurement, training, and influencing factors of working memory [3,4]. Visual working memory is the core system that supports individuals in completing various events in daily life and work. Visual working memory is a special system that processes, operates, and stores visual information by selecting a limited amount of visual information guided by visual attention [5]. Working memory capacity is divided into visual capacity and auditory capacity. Visual working memory capacity refers to the number of valuable objects that can be retained through visual observation [6,7]. There are many theoretical models of working memory, such as cognitive and neurobiological models [8] and the cognitive resource fixed model [9]. The most authoritative theoretical model of working memory is Baddeley’s [5], which has led to controversies on the influencing factors, processing, and training methods [10].

Many studies have shown that experts possess superior memory abilities in their respective fields, referred to as the cognitive advantages. Additionally, Mintzer illustrates that pictures are easier to encode than words in the memory process due to their cognitive advantages [11]. According to the literature, picture superiority denotes that pictures are more effective in enhancing memory compared to words when other factors are constant [12]. Previous comparative studies on picture superiority have utilized both words and pictures by similar participants, and the majority of the studies indicated that the subjects retained more information from the visuals [13,14,15]. The transfer effect refers to whether elite players have an advantage for items other than those with which they are familiar. In contrast, cognitive advantages entail comparative research between distinct groups of subjects. Hence, to facilitate the analysis, we used pictures to explore the cognitive advantages. Despite the several pieces of research on cognitive advantages [16,17,18,19], there are limited studies conducted to analyze the working memory cognitive benefits in elite athletes and novices. Is there an existence of working memory cognitive benefits among elite athletes? For instance, can professional players easily find relevant information in a game environment they are conversant with? Currently, studies on this advantage effect are increasingly comprehensive, and it is a trending investigation area to determine whether this effect exists in transfer.

Considerable findings have been made in the research on Visual Working Memory Capacity (VWMC). Ozimic’s research has revealed that the VWMC is primarily restricted by a representational system that enables the formation of independent visual object representations and an active maintenance system that enables the continuous activation of established representations even in the absence of external stimuli. Moreover, both these systems undergo transformations throughout an individual’s lifespan, and after reaching their peak at a particular age, they decline, resulting in a decrease in working memory [20]. Currently, numerous studies focus on the VWMCs of elite athletes [21,22,23]. Krenn’s study discovered differences in the executive function among elite athletes who participated in static, interceptive, and strategic sports. The findings reveal that strategic sports exhibit certain benefits over static sports regarding mean reaction times, cognitive shifting, and, to some extent, working memory [24]. Koch’s research suggests variations in the executive function among elite athletes. Furthermore, athletes in open-skill sports perform better in working memory and cognitive flexibility compared to those in closed-skill sports [25], a finding which was established by Holfelder’s research as well [26], while individual variations in the executive function of athletes were confirmed by Montuori [27]. Football is a team sport that calls for high cognitive ability from individuals during daily training tasks. Consequently, football players may exhibit various advantages in cognitive abilities [28]. Studying the VWMC cognitive advantages effect of elite football players is helpful in deeply exploring the processing process of the cognitive advantages effect and the key factors for its improvement. Mann’s study reveals that experts capture perceptual cues better than novices, as demonstrated by measurements of the response accuracy and response time [29]. Numerous factors affect VWMC, and research is currently focusing on identifying these factors. Yao’s research discovered that different visual working memory loads influence the hybrid search performance [30].

While these research studies can measure the VWMCs of subjects, they may be unable to provide a comprehensive overview of the VWMC features of diverse subjects in varying situational circumstances, hence the need for extensive research into cognitive advantage effects and transfer effects. Based on the aforementioned analysis, this study devised an experiment that employed three types of stimuli (professional, daily, and meaningless) as independent variables to observe the discrepancies in VWMC between novice and elite football players in diverse stimulus settings. Subsequently, the primary VWMC characteristics of elite football players under varying stimulus settings were examined, and the differences in the visual search ability between elite football players and novices were discussed through a reaction–time analysis. We hypothesize that elite football players benefit from a cognitive advantage effect, which is more pronounced in a professional setting and leads to a transfer effect, and which becomes more evident as the number of stimuli increases.

## 2. Subjects and Methods

### 2.1. Participants

An a priori power analysis using G*Power (version 3.1.9.7, Heinrich Heine University, Düsseldorf, Germany) was used to calculate the study’s required sample size. The parameters we choose were: (1) effect size f = 0.25; (2) α err rob of 0.05; (3) power of 0.95; (4) number of groups of 2. After calculation, a sample size of at least 72 can yield statistical significance. To be more conservative, we decided to recruit by including a minimum of 10% more participants than required. Ninety-nine participants were recruited for this study. Based on Swann’s proposed criteria for elite athlete classification [31], those who met the classification model were referred to as elite and semi-elite athletes; in this study, we refer to semi-elite and elite athletes collectively as elite athletes. Those classified as novices in the model were referred to as novices. The participants were divided into two groups: elite football players and novices. The present study was a single-blind study aimed at exploring the variance of VWMC between elite football players and novices. While the elite footballers were sourced from a sports university, the novices were selected from a university, excluding Physical Education majors.

**Figure 1:**
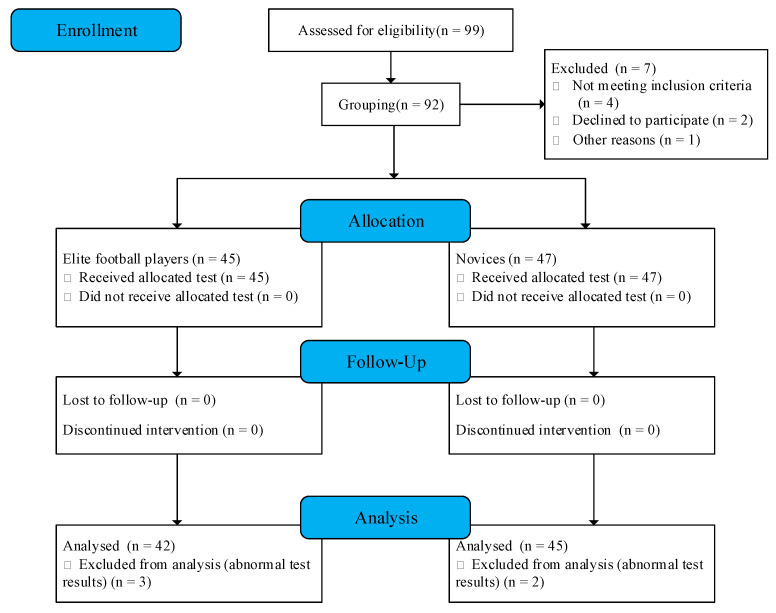
Flow diagram.

When the participants were recruited, oral and written notices were provided and the participants were asked to sign an informed consent form. Among the recruited participants, if one of the following conditions is present, they will be excluded: (1) suffering from physical diseases; (2) suffering from mental diseases; (3) suffered serious accidental injury within one year (such as fracture); (4) received similar experiments within the last year. An additional exclusion criterion was imposed if the experiment was not carried out as prescribed, or, if the test results were significantly abnormal, the study was tested at the recruiting universities and documented. After screening, 87 participants were included in the final analysis, consisting of 42 elite football players and 45 novices. The sample size used in this study was sufficient for the research objectives. The detailed screening situation was shown in Figure 1. The study was conducted in accordance with the Declaration of Helsinki and approved by the Ethics Committee of Northeastern University.

### 2.2. Experimental Materials

Experimental materials: The VWMCs of the participants were mainly measured using a classical change detection task [8], used in the experimental paradigm. The stimulus type was changed in line with the research purpose. The square stimulus in the original test was substituted with material corresponding to the research aim. Among them, under professional conditions, professional pictures were used as stimulation materials. Since elite football players were selected, the stimulation material of this experiment included the familiar pictures of football players as stimulation material. The selected professional stimulus materials were football-related items. Items such as selected football shoes, football clothes, leg guards, goals, scoreboards, whistles, flags, etc. Simple and familiar stimuli such as a desk, water cup, sofa, bed, TV, chair, dining table, and wardrobe were used in the daily condition. In meaningless conditions, the stimuli were the same as the classical task paradigm and process, nine different colors of 50-pixel squares were used, and the colors used were red (255, 0, 0), orange (255, 165, 0), yellow (255, 255, 0), magenta (255, 0, 255), cyan (0, 255, 255), blue (0, 0, 255), green (0, 255, 0), black (255, 255, 255), and white (0, 0, 0). The pictures under the selected professional condition and daily condition were all processed into 50 × 50-pixel square pictures using Photoshop 2021 (Adobe Systems Incorporated, San Jose, CA, USA).

### 2.3. Experimental Design

**Figure 2:**
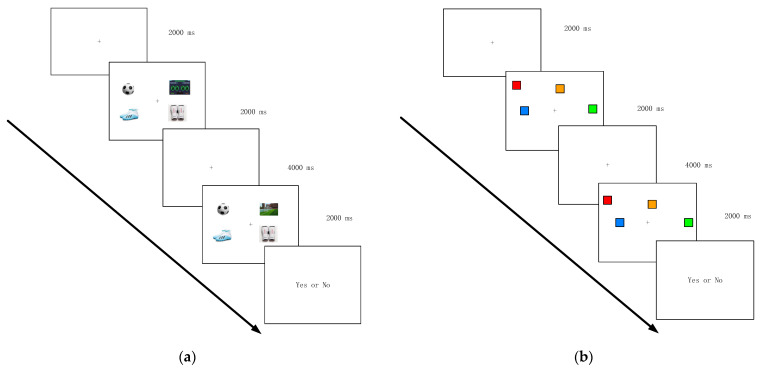
Experimental Design: (**a**) Examples of stimulus materials under professional condition; (**b**) Examples of stimulus materials under meaningless condition.

The experiment employed a 3 (stimulus type: professional, daily, meaningless) × 2 (group: elite football players, novices) two-factor mixed experimental design. The stimulus type was the within-group factor, and the group was the between-group factor. The experiment was programmed using E-prime3.0 software, the computer presented the experimental program, and a standard external keyboard was used for the key response. The study’s experimental paradigm uses the classical change detection memory task. In the specific experimental design, professional pictures replaced the square stimulus as the stimulus material under the professional condition. Under the daily condition, furniture pictures, which are more familiar to individuals, replaced the square stimulus. The meaningless condition involved block stimulation. Memory material replacement enables verification of the existence of cognitive advantages in VWMCs of elite football players. Lateral comparison between the memory material replacement experiments can also verify if there is a transfer effect of the superiority of elite football players. The test was conducted in the morning, and the participants were requested to rest as much as possible and perform at an average level the day before the test. The experiments were carried out in a quiet room. During the experiment, the subjects were required to keep their eyes fixed on the center of the screen. The testing sequence comprises three steps: conducting the test under professional conditions, then under daily conditions upon completion, and finally under meaningless conditions. Initially, a blank screen was presented on the computer for 500 ms, followed by the presentation of a fixation point “+” in the center of the screen for 2000 ms. Then, the center of the screen became the point of focus, and a stimulus matrix randomly appeared around it. The stimuli were required to be separated by a minimum of two degrees of the viewing angle and to not overlap each other. Under the professional condition, 2, 4, 6, and 8 football stimuluses were randomly selected and appeared within a 900 × 900 area (Figure 2a). Under the daily condition, easily distinguishable living objects in 2, 4, 6, and 8 were randomly selected from life stimuluses and presented in a 900 × 900 area. Under the meaningless condition, among the nine colors, randomly select 2, 4, 6, and 8 colors to appear in the 900 × 900 area (Figure 2b). After a delay of 4000 ms, and 2000 ms following the appearance of the memory stimulus array, the participants were required to respond promptly and accurately via keystrokes to discern whether the discovered matrix corresponded to the previous memory matrix. If they were the same, they pressed the A key, and if they were different, they pressed the L key. No matter whether it was the professional, daily, or meaningless condition, each experiment included a practice phase. There were 10 trials in the practice phase, 80 trails in the formal experiment, and 20 trails under the 4 different stimulus numbers. In the practice phase, when the subjects were familiar with the experimental process, the experiment starts. If not, they continued to practice until they did it. In the final results, it was found that the test data of three people in the elite athlete group were abnormal, and the test data of two people in the novice group were abnormal too, so the final analysis was excluded (the false alarm rate was too high). All the experiments were organized by professional psychology teachers, and no adverse reactions were reported during the experiment.

### 2.4. Statistical Analysis

SPSS 23.0 (IBM, Armonk, NY, USA) was used for the data analysis. To begin with, all the means and standard deviations of the data were evaluated using standardized statistical procedures. The normal distribution of the data was assessed using the Shapiro–Wilk test, and the homomorphic distribution was evaluated using Levene’s test. The effect sizes for the significant main effects and interactions were calculated using Partial Eta squared ($\eta_{p}^{2}$). To evaluate the variance in the different measurements, Mauchly’s test of sphericity was utilized. When the sphericity test was not satisfied, Greenhouse–Geisser analysis was conducted. Among them, the main analysis index was the K value, and the calculation formula of the K value was: K = stimulus item × (hit rate − false alarm rate). A secondary indicator of the analysis was the reaction time. All the tests were performed on the same day. MANOVA was used to compare the K values and reaction time of the elite football players and novices. The significant differences were expressed as *p*-values, where *p* < 0.05 was considered a significant difference.

## 3. Results

### 3.1. Participant Characteristics

The relevant demographic information was shown in Table 1, including age (F(1,85) = 0.674, *p* = 0.431), gender (χ^2^(1) = 0.778, *p* = 0.974), body height (F(1,85) = 0.573, *p* = 0.323), body weight (F(1,85) = 1.206, *p* = 0.167), education time (F(1,85) = 1.112, *p* = 0.265). It was shown that there was no significant difference between the two groups, indicating good consistency.

### 3.2. Comparison of VWMC under Different Stimulus Conditions

The K-value was calculated for varying stimuli conditions, and then a one-way ANOVA was used to compare the difference in the K-maximum value and K-mean value between the elite football players and novices under different stimulation conditions (professional, daily, meaningless). The results showed (Table 2): Under the professional condition, the K-maximum value (F(2,84) = 13.142, *p* < 0.001) and the K-mean value (F(2,84) = 12.095, *p* < 0.001) were both higher for the elite football players compared to the novices. Under the meaningless condition, the K-mean value of the elite football players was higher than that of the novices (F(2,84) = 5.326, *p* = 0.003). However, under the daily condition, the K-maximum value (F(2,84) = 1.789, *p* = 0.437) and K-mean value (F(2,84) = 1.642, *p* = 0.523) of the elite football players were not compared with the novices’ significant difference.

**Figure 3:**
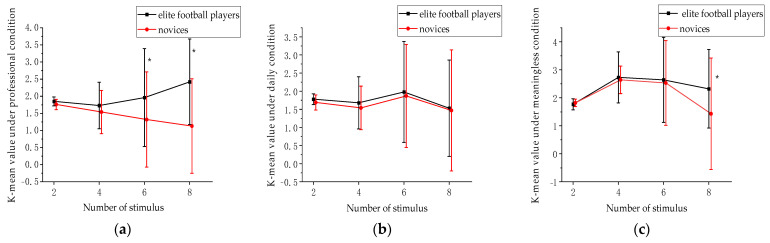
K-mean value between stimulus quantity and group (* *p* < 0.05; error bars: standard deviation); (**a**) under professional condition; (**b**) under daily condition; (**c**) under meaningless condition.

The changes in the VWMCs under different stimulus conditions were further analyzed with stimulus quantity, stimulus type, and the group as independent variables. The results showed that (Table 3): the main effect between the groups was significant, F(3,292) = 5.48, *p* < 0.001, $\eta_{p}^{2}=0.715$. The K-mean value of the elite football players was significantly higher than those of the novices in the professional condition (*p* < 0.001) and in the meaningless condition (*p* < 0.05), but not in the daily condition (*p* = 0.817). The main effect of the stimulus quantity was significant, F(9,866) = 7.97, *p* < 0.001, $\eta_{p}^{2}=0.514$, under different stimulation conditions, increasing the stimulus quantity, the K-mean value decreased significantly (*p* < 0.05). The interaction between the group and stimulus quantity was significant, F(9,866) = 3.27, *p* < 0.05, $\eta_{p}^{2}=0.261$; further simple effects analysis (Figure 3) showed that the elite football players had the most significant K-mean value advantage when the numbers of stimulus were both 8 (*p* < 0.001) in both the professional and meaningless conditions. There was no difference with the novices under daily condition (*p* > 0.05).

### 3.3. Comparison of Reaction Time under Different Stimulus Conditions

**Figure 4:**
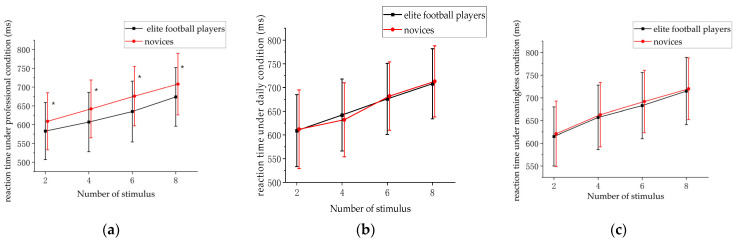
Reaction times between stimulus quantity and group (* *p* < 0.05; error bars: standard deviation); (**a**) under professional condition; (**b**) under daily condition; (**c**) under meaningless condition.

In order to further compare the differences in VWMC under different stimulus conditions, the subjects’ reaction times were selected for analysis. The reaction times of the subjects under different stimulus quantities were calculated, and then MANOVA was performed for analysis. The differences in the reaction times between the elite football players and novices under different stimulus conditions were compared. In various conditions, including professional, daily, and meaningless conditions, both elite football players and novices displayed quicker reaction times when they were faced with two stimuli. In other words, the less stimuli, the shorter the reaction time. For the elite football players, the average reaction time under the professional condition (603.32 ± 59.17 ms) was lower than that of the novices (684.90 ± 68.38 ms). However, the average reaction time of the elite football players (627.83 ± 63.19 ms) was not significantly different from the novices (635.32 ± 62.18 ms) under the daily condition. The results showed that the main effects between the groups were significantly different under the professional condition, F(2,188) = 8.73, *p* < 0.001, $\eta_{p}^{2}=0.313$. The reaction time in the meaningless condition (673.54 ± 60.53 ms) was significantly greater than that in the daily condition (632.15 ± 69.03 ms) and the reaction time in the professional condition (625.15 ± 58.23 ms), *p* < 0.001. Under the professional condition, when the number of stimuli was six, the difference between the reaction times of the elite football players (612.35 ± 64.32 ms) and that of the novices (693.21 ± 69.30 ms) was largest (Figure 4).

## 4. Discussion

This study aimed to investigate the variances in VWMCs between elite football players and novices using a modified change detection task. Various stimuli conditions were employed to provide a comprehensive analysis of the differences in VWMC between elite football players and novices under three different conditions. The VWMC was eventually confirmed and analyzed in conjunction with the reaction time measure. Research has shown that elite football players have a cognitive advantages effect on their VWMC. By comparing VWMCs under daily conditions, it was found that there was no significant difference between the elite football players and novices. However, by comparing the VWMCs of elite football players and novices under meaningless conditions, it was found that elite football players not only have a cognitive advantage in VWMC, but also may have a transfer effect. The findings were consistent with the hypothesis.

The VWMCs of the elite football players under professional conditions were significantly higher than those of the novices, which indicated that, in the memory task of professional information, the VWMCs of the elite football players had a significant advantage. Dodwell demonstrated that aerobic exercise could significantly improve VWMCs [32]. Elite football players have been shown to have an advantage in VWMCs that is attributed to their extensive exercise regime. This advantage is consistent with the transfer effect of VWMCs seen in elite football players. One possible explanation for this phenomenon is that participating in football as a sport can potentially improve individual VWMCs [33]. Another explanation was that, because of the individual differences in working memory [34,35], football players who were good at VWMCs may be better at competition than the football players who were not good at VWMCs, so they can improve to a professional level. The study found that the K-mean value of the VWMCs of the elite football players was higher than that of the novices under meaningless conditions, indicating that the advantage in the VWMCs of the elite football players was not only limited to memory tasks under the professional condition but also exists in general meaningless memory tasks. However, there was no difference between the elite football players and novices in the measurement of VWMCs under daily conditions. The possible reason was that, for the novices and elite football players, when the memory object was a familiar item, both the elite football players and novices produce the same response and therefore do not differ [36,37]. It may also be that the elite football players were more familiar with the types of stimuli under daily conditions than the students, so their processing processes were more complicated, and the corresponding visual working memory ability was also weaker. In terms of the professional and daily conditions, the professional and daily conditions were more detailed than the stimuli of the meaningless condition and were more difficult to identify than the meaningless condition. The elite football players maintained a certain degree of advantage in the professional condition and meaningless conditions, and there was no significant difference between them and the novices in the daily condition [38]. Since there was no cognitive advantage for the elite football players in the daily condition, we concluded that the transfer effect was not universal, and, in our study, the transfer effect existed only in the professional and meaningless conditions; the universality of the transfer effect will need to be investigated in depth in future studies. It cannot be simply explained by the general advantage of the elite football players in memory ability why they perform better than novices under meaningless conditions. The advantage does not appear in the daily conditions. Our study indicates that professional stimulation is more likely to benefit players under professional conditions. The advantage observed under meaningless conditions might be an indirect enhancement effect of professional stimulation on the general memory ability, also known as the transfer effect. Upon analysis of the K-maximum value of the elite football players and novices, under different stimulus conditions and different stimulus quantities, it was found that elite football players had the greatest advantage with a K-maximum value of 8 under professional conditions, 4 under meaningless conditions, and 6 under daily conditions. There was no significant difference in the K-mean value between the elite football players and novices under daily conditions, indicating that the elite football players do not have their own memory advantages. The possible reason was that, for the elite football players, the memory advantage was more dependent on their higher information processing efficiency in everyday conditions. Hein’s research shows that the working memory content can have an impact on subjects’ communication processes [39]. This further explains the differences in the VWMCs under different conditions. Therefore, under the professional condition, the elite football players have the most significant advantage when the number of stimuli was 8. However, when the number of stimuli was smaller, there was little difference with the K-mean value of the novices. For this difference, there may also be a relationship with visual attention. Lin demonstrated the effects of the object-based attention on the visual working memory [40]. From the interaction effect, under professional condition, the elite football players showed a significant memory advantage when the stimulus quantity was the largest, indicating that the memory capacity of the elite football players comes from being able to process the stimuli with larger numbers of objects at the same time. This also indirectly indicates that the elite football players may have larger attention spans and can allocate attention to more visual objects in a short period of time [41,42].

In this paper, by analyzing the reaction times, we can explore the memory processing process of the subjects. The longer the reaction time is, the greater the cognitive demands of the subjects on the current task are, which in turn affects the judgment time. The results showed that the reaction time showed differences in different tasks. In group comparison, the reaction time of the elite football players was lower than that of the novices, which proves that the elite football players maintain a large capacity advantage when performing memory tasks, which may be because their processing efficiency was high, and they have not yet reached the applicable limit of their cognitive resources in the current task. However, this study also has shortcomings. In terms of the selected subjects, this study selected athletes from open-skill sports. Closed-skill sports athletes were not selected. This paper did not compare the VWMCs of elite players in different sports. Moreover, there was no comparative study for gender or age in our study. Therefore, the differences in the VWMCs of elite players in open-skill sports and closed-skill sports can be compared in a future study. Investigating the disparities among elite football players with regard to their age and gender will also be a focal point of our forthcoming research.

## 5. Conclusions

Compared with the novices, the elite football players have more prominent VWMC cognitive advantages and transfer effects, which were verified to varying degrees in the different contextual tasks. Through further analysis of the reaction times, the cognitive advantage differences were found between the elite football players and novices not only when responding in the professional condition but also when responding in the meaningless condition.

## Figures and Tables

**Table 1:** Participant characteristics (M ± SD).

	Elite Football Players	Novices	*p*
Age (years)	20.46 ± 1.49	21.15 ± 1.69	0.431
Gender (male/female)	30/12	32/13	0.974
Body height (cm)	175.23 ± 3.13	176.48 ± 2.12	0.323
Body weight (kg)	65.32 ± 6.14	68.25 ± 7.13	0.167
Education time (years)	11.23 ± 1.22	10.21 ± 1.13	0.265

Note: M, mean; SD, standard deviation.

**Table 2:** VWMCs under different stimulus conditions (M ± SD).

	Professional		Daily		Meaningless	
	K-Maximum Value	K-Mean Value	K-Maximum Value	K-Mean Value	K-Maximum Value	K-Mean Value
Elite football players	3.68 ± 1.03	2.51 ± 0.56	2.90 ± 0.92	1.84 ± 0.62	3.21 ± 0.85	1.84 ± 0.67
Novices	3.09 ± 0.92	2.09 ± 0.66	2.95 ± 0.87	1.82 ± 0.55	2.53 ± 0.90	1.35 ± 0.59
*p*	0.000 *	0.000 *	0.437	0.523	0.002 *	0.003 *

Note: M, mean; SD, standard deviation; * *p* < 0.05.

**Table 3:** Comparison of K-mean value under different stimulus conditions and quantities (M ± SD).

Stimulus Quantity	Stimulus Condition	Elite Football Players	Novices	*p*
2	Professional	1.85 ± 0.13	1.76 ± 0.15	0.102
	Daily	1.78 ± 0.15	1.69 ± 0.21	0.167
	Meaningless	1.77 ± 0.20	1.82 ± 0.13	0.203
4	Professional	1.73 ± 0.68	1.54 ± 0.63	0.074
	Daily	1.68 ± 0.72	1.54 ± 0.60	0.215
	Meaningless	2.73 ± 0.91	2.64 ± 0.49	0.113
6	Professional	1.96 ± 1.43	1.32 ± 1.39	0.021 *
	Daily	1.98 ± 1.39	1.87 ± 1.42	0.226
	Meaningless	2.64 ± 1.52	2.53 ± 1.51	0.325
8	Professional	2.42 ± 1.25	1.13 ± 1.38	0.000 *
	Daily	1.53 ± 1.33	1.47 ± 1.67	0.136
	Meaningless	2.32 ± 1.40	1.43 ± 1.99	0.002 *

Note: M: mean; SD: standard deviation; * *p* < 0.05.

## Data Availability

The data presented in this study are available on request from the corresponding author. The data are not publicly available due to privacy reasons.
